# Recruitment and Resilience of a Harvested Caribbean Octocoral

**DOI:** 10.1371/journal.pone.0074587

**Published:** 2013-09-06

**Authors:** Howard R. Lasker

**Affiliations:** Department of Geology and Graduate Program in Evolution, Ecology and Behavior, University at Buffalo, Buffalo, New York, United States of America; University of Genova, Italy, Italy

## Abstract

Disturbance events are an important component of the ecology of coral reefs and increasingly frequent disturbances coupled with a lack of population resilience may contribute to changes in the structure of coral reef communities. The harvest of the Caribbean octocoral *Antillogorgia elisabethae* provides an opportunity to explore the relationship between adult abundance and recruitment and the manner in which recruitment contributes to the resilience of local populations. Recruitment of *A. elisabethae* was monitored in 20, 1-m^2^ quadrats at 8 sites along the southern edge of the Little Bahama Bank from 2004 through 2007. *A. elisabethae* has been harvested in The Bahamas for over fifteen years and all of the sites had been harvested three times, including a harvest during the course of the study. Abundances of adult colonies at those sites as well as a location that had not been harvested were also determined. Recruitment was highly variable, differing between sites, transects within sites, and, depending on the site, between years. Recruitment was best correlated with adult abundance averaged across the surrounding site. Regression analyses suggest abundance on smaller scales had only small effects on recruitment. The effects of the harvesting were site specific ranging from a 38 to 67% reduction in the density of mature colonies. The sites with the most abundant *A. elisabethae* continued to have the highest abundances after harvesting and there was no significant difference in recruitment before and after harvesting. Population size-structure at 6 of 8 sites that have been harvested multiple times exhibited an overall depletion in small colonies suggesting long term suppression of recruitment and declining populations. Severe depression of adult abundances coupled with local recruitment can create a negative feedback and lead to the decline of local populations. Populations that are dependent on self-recruitment are not resilient to large disturbance events.

## Introduction

Caribbean coral reefs have been subject to an unprecedented array of mortality events over the past 30 years, which have dramatically reduced the abundance of corals across many reefs habitats and sites [1], [2]. Coral reefs have always been subject to catastrophic mortality, and events such as hurricanes were considered part of a regular cycle of disturbance and recovery on Caribbean reefs [3]–[6]. Bleaching and disease events have added to the frequency of mortality on reefs, but the primary difference between the historical record and that of the past few decades has been the failure of reefs to recover from mortality events [7], and recruitment failure has been an important component of the observed patterns [8]–[10]. Recruitment failure or the inability of recruitment to compensate for increasing mortality may be an important factor in declining benthic populations [11]–[15]. Understanding the processes that control recruitment and how recruitment responds to disturbance takes on added importance as we attempt to discern the processes acting on contemporary coral reefs. Such studies may be particularly important for octocorals, as at least in some areas they appear to be increasing in abundance coincident with the decline in scleractinians [16]. Here I report on the recruitment and distribution of the Caribbean octocoral *Antillogorgia elisabethae* at sites in the Bahamas, comparing recruitment and local abundance, and how *A. elisabethae* populations have responded to the commercial harvest of the species at sites along the Little Bahama Bank.

Studies of recruitment over the past 20 years have shifted the paradigm from a belief that dispersal distances are great and that recruitment does not limit populations to one in which dispersal is often restricted and recruitment can limit population growth [17]–[19]. Dispersal is greatly influenced by reproductive strategy, with species that brood larvae exhibiting genetic and/or recruitment patterns indicative of less dispersal than broadcast spawning species among both scleractinians [20] and octocorals [21], [22]. In species such as the Mediterranean octocoral *Corallium rubrum* brooded larvae have extremely limited dispersal [23], [24] and recruitment rates among brooders such as the Caribbean scleractinian *Siderastrea radians* are affected by density of adults in the surrounding habitat [25]. If recruits at a site are local in origin, then adult abundance may control recruitment rates and populations with these traits may have less resilience to local disturbance events, especially when those events occur frequently. The harvest of *A. elisabethae* on the Little Bahamas Bank provides opportunity to determine how the artificial reduction in the density of reproductive colonies has affected local recruitment and the resilience of populations.

Recruitment has not been considered the life stage most affecting population growth among reef taxa [11], [12], [21], [26]–[28], but resilience from disturbance may be more dependent on recruitment. Linares et al. [13] reported the effects of a mass mortality event on populations of the Mediterranean gorgonian *Paramuricea clavata. P. clavata* is a surface brooder, and they noted that recruitment did not keep up with the increased mortality that occurred following the mortality event. In subsequent analyses, they found that, if recurrent, the events would almost inevitably lead to local population extinction [29].

*A. elisabethae* [30] is patchily distributed through much of the Caribbean. (Caribbean species previously classified as *Pseudopterogorgia* spp. have recently been assigned to the resurrected genus *Antillogorgia* [31].) When present, *A. elisabethae* and its congener *A. bipinnata* are among the most common Caribbean gorgonians at intermediate (10–25 m) depths (Belize [32], San Andres [33], Jamaica [34]). In The Bahamas the species has high abundances in some areas but is often absent in adjacent, seemingly similar habitats. The basic life history of the species has been described by Gutiérrez-Rodríguez and Lasker [35]. Colonies are gonochoric and mature at approximately 15–20 cm height. Female colonies surface-brood embryos, which are washed off of colonies usually after several days, at which time the planulae are negatively buoyant and competent to settle. Gutiérrez-Rodriguez & Lasker [35] followed released planulae and observed that 13% fell to the substratum within 5 m of the natal colony. Thus *A. elisabethae* is likely to exhibit some level of philopatry, which may be evident in the spatial distribution of colonies. The species has been commercially harvested for a natural product, pseudopterosins, for over 15 years [36]. During that time period collectors have cropped tissue from *A. elisabethae* colonies, leaving behind a 5–15 cm tall colony containing about 5–10 branches. Sites along the Little Bahamas Bank have been harvested up to 4 times. Most colonies cropped in the harvest apparently recover, but survival rates are unknown and some mortality among cropped colonies has been observed [37]. The primary branches of cropped colonies exhibit increased growth rates [36] and polyps throughout the colony have reduced fecundity [38]. Overall the harvested colonies will have dramatically lower reproductive output compared to their pre-harvest size due to the loss of most branches. The likelihood that *A. elisabethae* has philopatric larvae, the species’ natural variability in abundances and the reduction in reproductive output likely to occur following harvests makes this species an excellent system to assess the extent to which recruitment is constrained by local abundance and to assess the resilience of populations from local disturbance events.

## Methods

**Figure 1:**
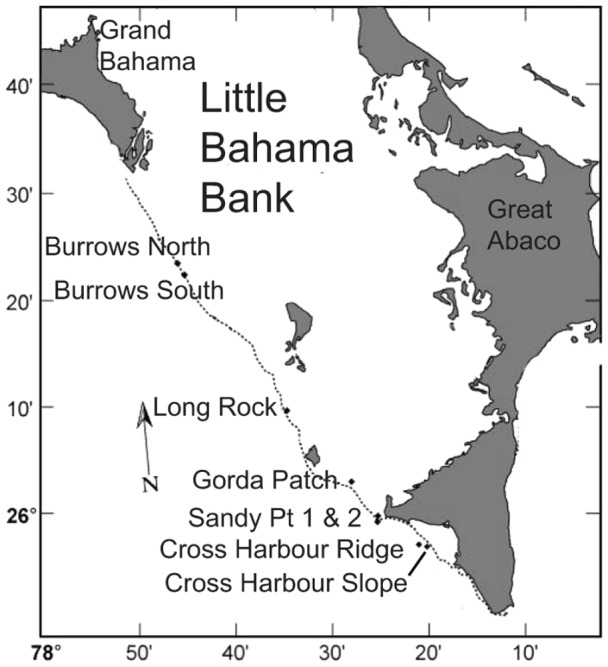
Map of the Little Bahama Bank showing the location of the study sites.

The spatial distribution, population size-structure and recruitment of A. elisabethae was assessed at 8 sites along the southern edge of the Little Bahama Bank (Fig. 1) between Burrows Cay and Great Abaco Is. Censuses of adult colonies were conducted in Nov 2004 and repeated in November 2005 and January 2007. Recruitment was assessed 5–6 months and again 11–12 months after spawning in May and November in 2004 and 2005, in June 2006 and then in January and May 2007. The sites chosen for the study were selected to be a representative sample of harvested sites. They all had had sufficiently high adult colony densities that they were subject to harvest. Within that constraint they were chosen to span a range of densities, and had preliminary density estimates that ranged from 0.45 to 8.95 colonies >5 cm tall per m^−2^. The sites ranged in depth from 8 m (Gorda Patch Reefs) to 22 m (Cross Harbour Ridge), which covers the range over which the harvest occurs. All of the sites had been harvested 2–4 years prior to the initial surveys. Censuses and collections were made under a research permit granted by The Bahamas Department of Marine Resources.

At each site a “center point” was arbitrarily selected and a randomly determined compass heading and a random distance between 1 and 40 m were then used to establish the placement of a 20-m transect line which was then laid out on a second random compass heading. Four transects were established at each site. Five 1×1 m “recruit quadrats” were then randomly placed on each transect and permanently marked. If a randomly chosen quadrat did not contain any octocorals in November 2004, the point was considered inhospitable to octocorals and a replacement quadrat was randomly selected. This most commonly occurred when the entire 1-m^2^ area was covered by sand. Thus, while some recruit quadrats were devoid of *A. elisabethae*, all contained octocorals. Records of “rejected” quadrats were maintained and it was possible to compare data based on the original 5 quadrats with those from the 5 quadrats that contained octocorals. The basic layout of transects and recruit quadrats is diagrammed in Jamison and Lasker [22].

Additional data were collected between May 2004 and Nov 2005 at two sites that were selected due to the absence of *A. elisabethae*. At each of those sites 4 transects were established as described above. On two transects at each site, 50 large branches of *A. elisabethae* were suspended above the substratum by inserting the basal end of the branch into a 10-m length of braided polypropylene rope which was suspended between two posts approximately 1 m above the substratum. The branches were collected from large colonies at the closest location in which there were abundant *A. elisabethae.* The colonies were large enough to produce gametes. Quadrats at the sites were searched for *A. elisabethae* recruits in May and November 2005.

### Population Abundance

During each census the number and heights of A. elisabethae colonies in each recruit quadrat as well as those in each of the 8 adjacent 1×1 m^2^ blocks (referred to henceforth as near neighbors) were also determined. Enumerating recruits requires extremely careful surveys, and only colonies >5 cm were counted in these censuses. The design measured abundance of established colonies at the scales of quadrats, near neighbors, transects (up to 45 m^2^ of area along each line) and sites. Since the randomly placed recruit quadrats on a transect were sometimes next to each other or spaced 1 m apart, the groups of 8 near neighbor 1-m^2^ areas sometimes overlapped. Estimates of absolute density, which include those quadrats initially scored as “inhospitable” due to the absence of any gorgonian species were conducted at the levels of quadrats, transects and sites. Two sites, Sandy Point 1 and Sandy Point 2, were not censused in 2004, and only censuses after a 2005 harvest were obtained.

In order to develop an estimate of A. elisabethae density on a larger scale a series of belt transects were also surveyed around each site. Ten 20×2 m belt transects were surveyed and heights all A. elisabethae were recorded. Locations of transects were selected by randomly choosing latitude and longitude values (+/−0.01′) within 1 km of each site. Only sites <25 m depth were surveyed. Most of the >25 m sites were far deeper than 25 m or were sand substrata, and densities for those belt transects that would have been below 25 m were scored as zero. For instance, as the name implies, Cross Harbour Ridge was located on a ridge that dropped steeply to the south of the site and many of the randomly selected positions for belt transects at that site were in those deeper (>>50 m) waters.

Only reproductively mature colonies were considered in comparisons of population density and recruitment. The smallest reproductive colony found in surveys by Gutiérrez-Rodríguez and Lasker [35] was 18 cm height. In this analysis, only colonies 20 cm in height and taller were classified as adult. While some slightly smaller colonies may have been reproductive [38], those colonies due to their small size would only produce a small number of eggs [39]. Larval production within a population should be a function of the aggregate branch length of all of the colonies, not simply the number of colonies. In A. elisabethae total branch length, which I shall refer to as adult area, is related to square of the colony height [40]. The sum of adult areas of all colonies, henceforth cumulative adult area, is the sum of the squared height of all colonies that were >20 cm tall. Adult density (colonies m^−2^) and cumulative adult area (cm^2^ m^−2^) were both used as indices of adult abundance.

Size frequency data from two 20×1 m belt transects at a site near Wood Cay, Grand Bahama, where there was no evidence of a previous harvest, were collected in June 2012. Those data were used for comparison with harvested sites.

### Recruit Collections

In the northern Bahamas, *A. elisabethae* spawns in November and December (or early December and January) following the new moon [35]. An initial collection of the recruits that had settled in 2003 was made in May 2004. Growth rates of small (<10 cm height) colonies are typically 4 cm y^−1^ [41]. Thus colonies from the 2003 spawning event were most likely less than 5 cm in height, and all colonies <5 cm in height in the initial collection were considered to be recruits from the 2003 spawning event. In the initial May 2004 census, recruits were approximately 3–5 cm tall and only those that were clearly *A. elisabethae* were censused. Recruits that had been missed in the May census were found in a November survey. In May 2005 we encountered large numbers of 2–4 cm *Antillogorgia* spp. recruits, which could not consistently be identified on the basis of gross morphology alone. Starting in May 2005 we collected all *Antillogorgia* spp. recruits, preserved them in 95% ethanol and identified the *A. elisabethae* using several microsatellite loci following the protocols of Jamison and Lasker [22]. Recruits were surveyed in that manner in May 2005, November 2005, June 2006, January 2007 and May 2007. The 2007 collections were made approximately 6–8 weeks after spawning (January) and then 5–6 months after spawning (May). The vast majority of recruits collected in 2007 were collected in January. Those recruits were only a few millimeters in height and had one or two polyps. Some larger recruits, which presumably had been missed in January, were collected in May 2007.

Collections after November 2004 included large numbers of *Antillogorgia* spp. recruits and at one of the sites, Cross Harbor Ridge, we regularly collected hundreds of recruits from single quadrats. Site-wide (i.e., 20 m^2^) totals of 1652, 1428 and 1063 recruits were collected in 2005, 2006 and 2007 respectively. Those collections included all *Antillogorgia* spp. In order to estimate the number of *A. elisabethae* recruits at Cross Harbour Ridge, subsets of recruits from each census (the recruits found in 10 arbitrarily selected recruit quadrats) were identified using microsatellites [22]. The year specific proportions of those samples that were *A. elisabethae* were then used to estimate numbers of *A. elisabethae* among the remaining samples. The proportion of samples that were *A. elisabethae* varied between the four years at Cross Harbour Ridge (Chi-square test of independence, p<0.001). The proportion of *A. elisabethae* recruits also differed between sites (Chi-square test of independence, p<0.001). A total of 338 samples from the other sites were not identified due to field mishaps and technical difficulties. The proportion of those that were *A. elisabethae* was estimated using site-specific proportions of *A. elisabethae.*

## Results

### *Antillogorgia elisabethae* Abundance Patterns

Densities of A. elisabethae at the start of the study and before the 2005 and 2006 harvests are presented in Table 1. The number of colonies within individual quadrats in November 2004 ranged from 0 to 14 (mean = 2.0+/−0.2 [std. error]) for immature colonies and 0–9 for mature colonies (mean = 1.1+/−0.2). At all but one site there was little difference between densities reported in Table 1 and those calculated when the sand covered quadrats were included in the analysis. Burrows North was characterized by coral mounds on a sandy bottom, and the calculated density of A. elisabethae was approximately 50% lower when quadrats that were all sand were included in the analysis. A. elisabethae had a patchy distribution with variation evident between sites, transects and between quadrats (Table S1). The distribution of variance at these three spatial scales was different depending on whether recruits, immature or mature colonies are being considered. Among recruits the greatest contributor to the total variance was at the level of transects (72.8%) with smaller and near equal variance added by quadrat and site level variation (13.8 and 13.4%). This suggests the “patch size” of recruitment most closely reflects the 20 m scale of the transects. Most recruits do not survive and thus densities and variances of immature and adult colonies are lower than among recruits (Tables 1 and S1). Transect level variation was over 5 times greater than between quadrat variation among the recruits but that pattern was reversed for both immature and mature colonies. Quadrat to quadrat variation accounted for more of the variance among the immature (55.9%) and mature colonies (75.3%) than transect scale variation (immature colonies, 11.2%; mature colonies, 2.2%). The increased role of quadrat and site level variance suggests that differences in survival operating on a finer scale was the dominant effect controlling the abundance distribution despite the transect scale variation created by the recruitment patterns. These patterns remained virtually unchanged even when the analysis was limited only to quadrats containing A. elisabethae (i.e., no 0 values).

**Table 1:** Density* of Antillogorgia elisabethae (colonies m^−2^) at 6 sites on the Little Bahama Bank in November 2004.

		All colonies		Recruits^1^ (≤5 cm height)		Immature (5.1–19.9 cm height)		Mature (≥20 cm height)		Proportion of adult colonies harvested^2^
	Depth(m)	Mean	(std. error)	Mean	(std. error)	Mean	(std. error)	Mean	(std. error)	
Burrow North	16	4.80	1.05	0.65	0.22	2.40	0.54	1.75	0.51	0.52
Burrow South	16	4.26	0.75	1.26	0.38	1.68	0.39	1.32	0.38	0.60
Long Rock	13	0.50	0.31	0.05	0.05	0.15	0.15	0.30	0.15	0.67
Gorda Patch Reef	6	3.85	0.58	1.30	0.33	1.75	0.34	0.80	0.25	0.50
Cross Harbour Slope	18	10.85	1.74	6.30	1.31	3.05	0.67	1.50	0.30	0.03
Cross Harbour Ridge	22	21.85	4.22	12.90	3.83	5.60	0.78	3.35	0.58	0.38

* Values are means (standard error) based on censuses of 20 1 m^2^ quadrats at each site. The values overestimate absolute density as only quadrats containing gorgonian colonies were included in the study. All sites had been harvested ≥2 years earlier. The Sandy Point 1 and Sandy Point 2 study sites were not surveyed prior to the most recent harvest and are not included.

1 Recruits were generated by the November 2003 spawning event and were 1 year old at the time of the census.

2 Proportion of adult colonies harvested is based on the change in >20 cm height adults in censuses conducted before and after the 2005/2006 harvests.

**Figure 2:**
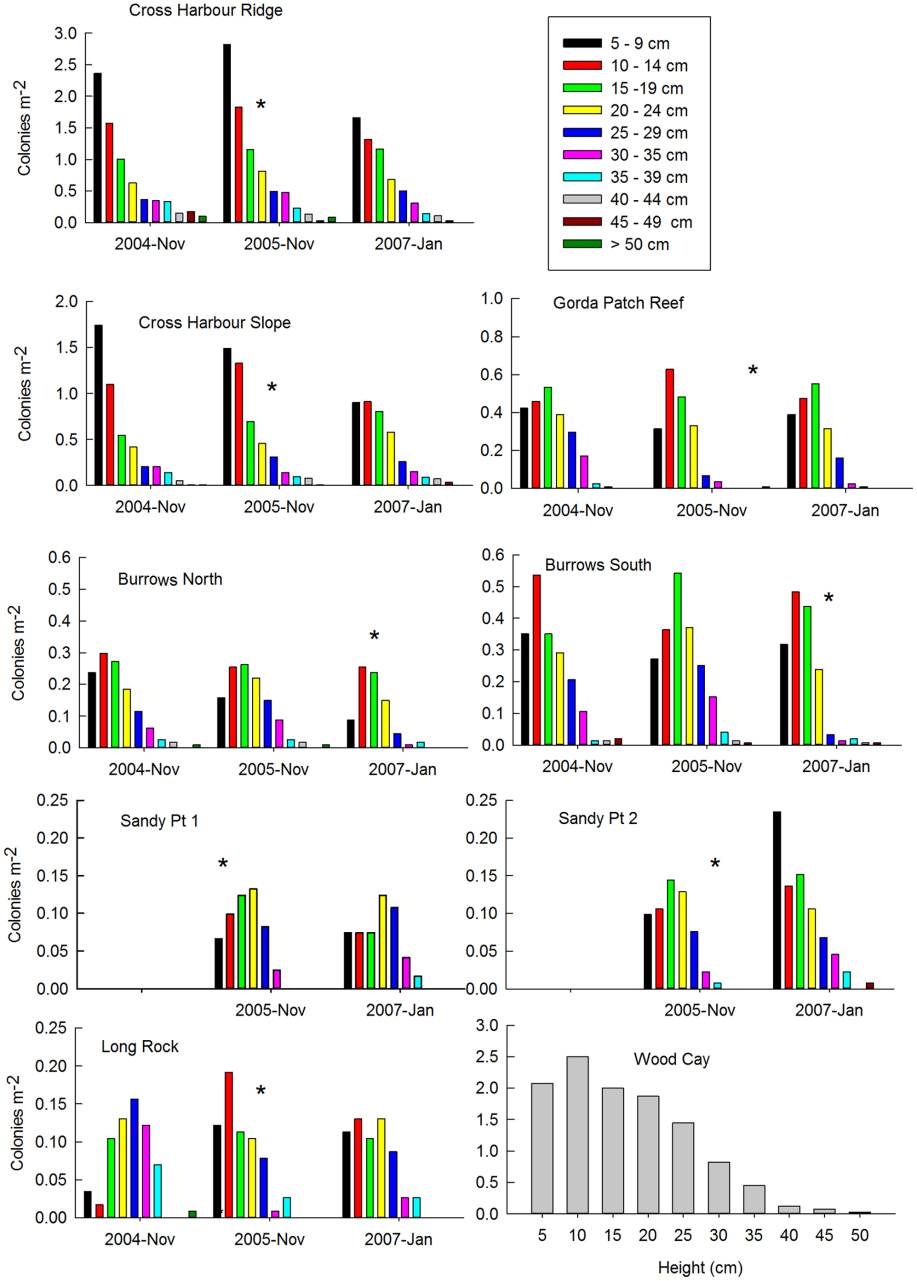
Size-frequency distributions of *Antillogorgia elisabethae* at sites in The Bahamas. The populations at Wood Cay had never been harvested. All of the other sites were harvested multiple times. Censuses completed within a year of the most recent harvest are denoted by an asterisk. Census data from the two Sandy Point sites were not collected prior to the 2005 harvest. Note the differences in scale between the different sites.

With exception of Cross Harbour Slope, all of the harvested sites had lower densities of adult (i.e. >20 cm tall) colonies following the harvests (Table 1). The size-frequency distributions of colonies at the sites (Fig. 2) illustrate the effects of both the harvest that occurred during the study and the longer term effects of multiple harvests. At most sites the year following the 2005/2006 harvest was characterized by a depression in the proportion of colonies greater than 25 cm height, and an increase in 10–14 cm and, to a lesser extent, 15–20 cm colonies. That presumably reflects the change in height of the cropped colonies.

Differences between sites and the longer term effects of harvesting are also suggested in the size frequency distributions. The Wood Cay population, which had not been harvested, had greater density of all size classes than most of the other sites and the population was dominated by smaller colonies (5–9 and 10–14 cm) than the harvested sites. The relative depletion of small colonies following the 2005/2006 harvest also appears to be greatest at the sites with the lowest density. Many of the sites already exhibited a relative reduction in small colonies in November 2004 and to some extent that reflected the time elapsed since the preceding harvest. The previous harvest at the Sandy Point sites was in December 1999, Gorda Patch Reef and the Cross Harbour sites in December 2001, the Burrows sites in January and June 2002 and Long Rock in June 2002.

### Recruitment

**Figure 3:**
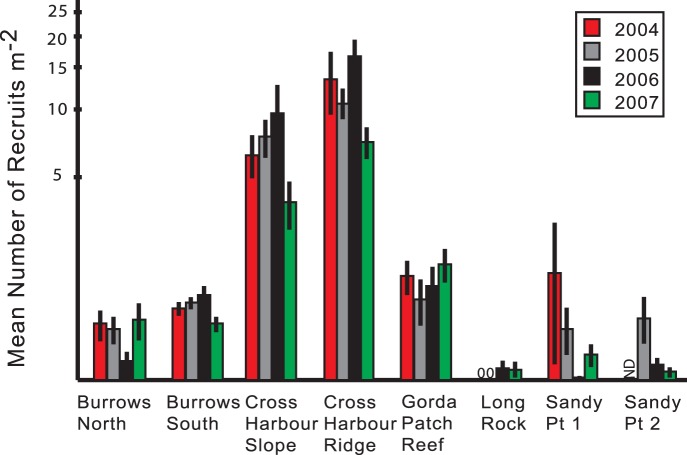
Average numbers of *Antillogorgia elisabethae* recruits found in 1 m^2^ quadrats at 8 sites on the Little Bahama Bank. Harvests conducted during the study would have affected recruitment in 2006 and 2007, except at the two Burrow sites where their effects would only appear in the 2007 censuses. ND, no data. Error bars are standard errors.

With the exception of Long Rock, A. elisabethae recruits were found at each site in each year, but recruitment was highly variable across sites (Fig. 3). Repeated measures ANOVA of recruitment with years as the repeated variable and transects nested within sites (Table 2) identified significant variation in recruitment between sites (p<0.0005), transects within sites (p<0.0005) and in the site by year interaction (p = 0.001), but variation between years was not significant (p = 0.179). Recruitment was low in 2007 at a number of sites, but as the significant interaction indicates, the effect was not uniform across all of the sites. Within any single site there were only significant (p<0.05) year to year differences at Cross Harbour Slope (2005 vs. 2007) and Cross Harbour Ridge (2004 vs. 2005, 2004 vs. 2006 and 2006 vs. 2007, post-hoc least significant difference tests among sites and years from a one-way analysis of variance in which data from each site and year were treated as independent observations). The 2007 estimates were based in large part on January censuses, and therefore did not include 3 months of post-settlement mortality that would have occurred prior to the May sampling conducted in other years. Thus the 2007 values may be inflated relative to the other years, which makes the lower values at some sites particularly notable. Post-hoc tests in pairwise comparisons based on an analysis that did not treat years as a repeated measure (Table S2) identified 4 homogenous groups, Cross Harbour Ridge, Cross Harbour Slope, a third group containing the two Burrows sites and the Gorda Patch Reef and a fourth group containing the remaining sites as well as the Burrows sites. As the error bars in Fig. 3 suggest the data were heteroscedastic even after transformation and the ANOVA alone cannot be interpreted as a definitive. After pooling the data by site or year, a Kruskal-Wallis Test for differences between sites, year and a Friedman Test for differences between years identified a significant site effect (p<0.0005) but no significant difference between years (p = 0.155).

**Table 2:** Repeated measures ANOVA of ln(1+recruits per quadrat) across site, transects within sites with year as a repeated measure (SPSS, v. 20, Mixed Model Analysis).

Type III Tests of Fixed Effects									
Source	Numerator df		Denominator df			F		Sig.	
Intercept	1		182.149			426.690		<0.001	
Site	7		125.137			120.846		<0.001	
Year	3		160.682			1.655		0.179	
Site * Year	21		147.289			2.368		0.001	
Transect(Site)	24		120.455			3.926		<0.001	
**Estimated Marginal Means**									
**Site**		**Mean** a **(ln[1+recruits])**		**Std. Error**	**Year**		**Mean** a **(ln[1+recruits])**		**Std. Error**
Burrows North		0.304		0.068	2004		0.642		0.105
Burrows South		0.358		0.608	2005		0.757		0.042
Cross Harbour Slope		1.611		0.068	2006		0.666		0.049
Cross Harbour Ridge		2.137		0.068	2007		0.630		0.042
Gorda Patch Reef		0.644		0.068					
Long Rock		0.036		0.068					
Sandy Point 1		0.187		0.068					
Sandy Point 2		0.114		0.189					

a Based on modified population marginal mean.

Recruitment rates were highest within those quadrats with the greatest abundance of adult colonies. Numbers of recruits found in each of the quadrats in each year were more closely correlated to cumulative adult area (i.e., the sum of the square of the colony heights) than to adult density (Pearson’s correlation coefficients, r = 0.43 vs. r = 0.33; 0.45 vs. 0.41 using (ln (x+1) transformed data, all values p<0.001) and all subsequent analyses used cumulative adult area as the measure of adult abundance.

**Figure 4:**
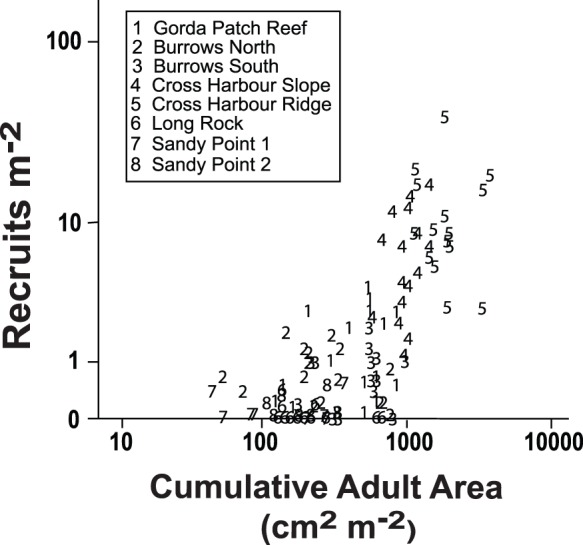
*Antillogorgia elisabethae* recruit density on each transect as a function of the abundance of adults measured as the cumulative colony area on the transect ( = Σ [colony height]^2^). The graph contains 16 points each for sites 1 through 6 and 12 points each for sites 7 and 8. Many points with low recruitment rate are obscured by overlapping data.

Recruitment to the individual quadrats was more closely related to adult cumulative area when adult cumulative area was averaged over larger scales. The relationship between recruitment and adult cumulative area was explored with stepwise linear regression using adult cumulative area at four scales, each quadrat, adjacent (near neighbor) quadrats, all quadrats on the transect, and all quadrats at the site as the independent variables. Adult cumulative area was calculated such that adult density data from the quadrat being analyzed was not included in the nearest neighbor estimate, data from the nearest neighbors not included in the transect and so on. Thus the four levels of adult cumulative area used independent data. When the measures of adult cumulative area were adjusted as described there were still significant correlations among the values from within the sites (Pearson correlation coefficients from 0.42 to 0.77, all p<0.001) but that colinearity among the independent variables was not due to shared data but rather the distribution patterns of the adults. The density of colonies on the belt transects surveyed within 1 km of the study site, labeled “Regional Density” was also included as an independent variable in the multiple regression. Regional Density was not correlated with any of the other measures of abundance. The relationship between recruitment and adult density is illustrated in Fig. 4.

Cumulative adult area at the scale of sites explained 46% of the variance; quadrats an additional 2% of the variance. All of the other variables significantly improved the regression but each explained less than 1% of the variance (Table S3). Regional Density had a negative relationship with the observed recruitment rate which underscores the point that the regression characterizes statistical associations and not necessarily causal relationships. (Given distance of the regional density transects to the recruitment sites, it is difficult to envision a mechanism for a negative density dependent effect.) The constant in the regression, i.e., the y-intercept, can be interpreted as a representation of a baseline level of recruitment that occurs independent of local adult abundance. The constant, 0.083, was not significantly different from 0.0 and the mean and 95% confidence interval correspond to recruitment rates of 0.08 recruits m^−2^ with a 95% confidence interval of 0.0 - 0.21 recruits m^−2^. Those values are only a small proportion of the 2.86 recruits m^−2^ mean recruitment rate observed across all of the sites and years. As the same quadrats were examined in each year and are not independent observations, the regressions were also repeated for each year. As in the analysis of the pooled data set, site level cumulative adult area explained the largest component of variation (>40% in all 4 years, data not shown) with adult area at smaller scales accounting for 5% or less of the variation in recruitment.

The experimental introduction of *A. elisabethae* branches at two sites had no clear effect on recruitment in the following year. *A. elisabethae* recruits were not observed at one site and only 5 recruits were found at the other site. At that site 3 of the five recruits were observed on transects containing the explant colonies and two from a single quadrat on one of the control transects, approximately 20 m from the nearest explant colonies.

### Effects of Harvesting on Recruitment

All of the eight sites were harvested during the course of the study. In some cases the harvest was apparent, due to the presence of colonies that showed evidence of being recently clipped, while at other locations, where harvests were less thorough, the inclusion of the site among harvested sites is based on reports from the collectors. Based on the surface GPS position that the harvesters used, the Cross Harbour Slope site was harvested in 2005, but there was no evidence of a recent harvest, which suggests the transects monitored in this study were missed when the area was harvested. The harvest decreased adult abundances at the other sites, but harvest efficiency, i.e., the change in abundance after harvesting, varied between sites. Efficiency at sites where there was evidence of the harvest ranged from 38% to 67% of the adult colonies. Harvest efficiency was low at Cross Harbour Ridge, where there was clear evidence of the harvesting, but after harvesting the site still had *A. elisabethae* densities that were greater than the pre-harvest densities at the other sites.

The effect of the most recent harvest on recruitment was assessed by comparing recruitment within quadrats between the years immediately before and after harvesting in a repeated measures ANOVA (2005 vs. 2006 for 6 of the sites and 2006 vs. 2007 for the two Burrows sites). There were insufficient sample sizes to include transects as a nested variable in the analysis and the analysis was conducted using quadrats as replicates within sites and then using average recruitment rates for each transect as the replicated value. In both analyses (Table S4) recruitment differed between sites (p<0.001, in both analyses), but there was no difference in recruitment before and after harvest (individual quadrats, p = 0.759; transect averages, p = 0.424).

## Discussion

The population structure and recruitment data from *A. elisabethae* provide insight into the effects of the harvest and on the broader questions of the relationship between local abundance and recruitment, and how that might affect the resilience of populations to disturbance. In general, the data indicate that adult abundance, and by extension local supply of planulae, affects recruitment rates. Although the single harvest that occurred during the study did not have a discernible effect on recruitment, some of the sites that have been harvested multiple times had low densities of mature colonies, and were characterized by low recruitment rates and disproportionately low abundances of small colonies.

Comparisons of recruitment data between studies are always difficult due to the diversity of methodologies used. Studies that have monitored octocoral recruitment within randomly chosen natural areas (*Leptogorgia virgulata* [21], *Paramuricea clavata* [42], *Plexaura kuna* [26]) have reported rates that are similar to those for *A. elisabethae*. Among those data there is no obvious difference between broadcast spawning and surface brooding species, but Jamison and Lasker [22] found lower recruitment rates among broadcast spawning *Antillogorgia* spp. in a comparison of 6 *Antillogorgia* spp. in 2005 from the two Cross Harbour sites included in this study. Studies that have used settling plates as substrata have generally reported much higher recruitment rates. Bramanti et al. [43], [44] examined recruitment of the Mediterranean octocoral *Corallium rubrum* on settling plates placed in habitats with abundant *C. rubrum*. Recruitment rates at their study sites were 10–100 times greater than the highest values reported here [43], which probably reflects the combined effects of using settling plates that provided a uniformly suitable settlement substratum, the proximity to spawning colonies and the extremely small 23–32 cm mean dispersal distances reported for *C. rubrum* [23]. Similarly settling plates set up around spawning *Heliopora coerulea* in the Ryukus Is., Japan accumulated up to 3.7 recruits 100 cm^−2^ [45], a density over two orders of magnitude greater than for *A. elisabethae.* The *A. elisabethae* data illustrate how limited larval dispersal can affect the resilience of populations to repeated disturbance. Philopatric recruitment of larvae can facilitate recovery of populations from small disturbance by providing a ready source of larvae, but it may reduce resilience if the disturbance is too great. For instance, Cupido [42] observed that a massive mortality event in the Mediterranean Sea in 1999 which led to the loss of 75% of the *Paramuricea clavata* colonies also was associated with reduced recruitment. The effects of harvesting of *A. elisabethae* populations were most evident in the absolute density of colonies and their size-structure. Unlike populations not subject to harvesting (i.e. Wood Cay and San Salvador [40]) many of the harvested populations had fewer small colonies (i.e. recent recruits) than large colonies both before and after the most recent harvest. Storage effects, the persistence of large, older individuals coupled with intermittently high recruitment [46], could generate patterns similar to those observed. However, although annual recruitment differed between some years at some sites, large annual variation in recruitment was not observed over the 4 year study, nor was there suggestion of episodic recruitment in the size distribution from Wood Cay. All of the sites followed in this study had dense *A. elisabethae* populations at the inception of the commercial fishery. The low density and skewed size distribution pattern at some of the sites probably reflects the cumulative effects of reduced populations and local dispersal on recruitment. The data from Long Rock are particularly striking as prior to 2004 the site supported one of the densest populations (T. Higgs, pers. comm.).

Harvesting is likely to have been the driving force in the patterns observed in the *A. elisabethae* populations, but the sites may also differ in either the supply of recruits from other locations, in the initial settlement and survival of available recruits or some other habitat difference affecting both recruits and established colonies. For instance, Gotelli [21] found that both recruit and adult abundances of the broadcast spawning *Leptogorgia virgulata* were correlated with habitat quality, a measure of available settlement substratum. Macroalgae are known to interfere with octocoral recruitment [21], [47], and differences in macroalgal cover, which was high at all of the *A. elisabetghae* sites, could at the least amplify the effects of reduced local production of planulae.

While data before harvesting commenced are not available from the sites, it is clear that all have not been affected equally. The two Cross Habour sites, still supported dense *A. elisabethae* populations and had the highest recruitment rates. Even after harvest these sites had more mature *A. elisabethae* colonies than any of the other sites (Table 1). The result suggests that high recruitment can be maintained at sites where harvesting leaves behind large standing populations and these populations have exhibited greater resilience to harvesting. However, when adult abundance is sufficiently depressed reduced recruitment and eventual population collapse can be expected.

In considering the effects of harvesting on *A. elisabethae* it is also important to note that the best predictor of recruitment was cumulative squared colony heights. This is not a surprising result as reproductive output of colonies will be a function of the number of polyps which is more closely related to the number of branches and that to the square of colony height [40]. An additional consequence of this relationship is that harvesting by cropping the largest colonies removes more of the population’s reproductive output than the simple decline in the number of reproductive colonies would suggest. For instance, in group of 24 *Eunicea flexuosa* colonies 98% of the eggs were produced by the 12 largest colonies [39], *Paramuricea clavata* exhibits a similar pattern [48]. This effect will only be exacerbated by the decline in per poly fecundity that occurs in harvested colonies [38].

While the harvest of *A. elisabethae* has generated somewhat mixed results, harvests of other, similar species have been quite successful. Grigg [49] found that harvesting of the deep-water octocoral *Corallium secundum* did not affect recruitment at a site in Hawaii, and similarly, has concluded that over two decades of annual harvesting of the black corals *Antipathes dichotoma* and *A. grandis* in Hawai’i did not affect recruitment [50]. Harvesting of these deep water species is made difficult, and probably less efficient, by the limitations on harvesting at depth. In contrast, exhaustive collections, achieved by thorough or frequent harvests, are more likely to adversely affect recruitment and the long term resilience of populations. Thus concerns for recruitment should be factored into management plans for *A. elisabethae* and similar species.

Although harvests of *A. elisabethae* have been conducted at a greater frequency than natural disturbances such as hurricanes, the harvest of *A. elisabethae* at sites in The Bahamas is similar to natural disturbances in that the effects are not uniform across all localities nor among all size classes [51], [52], and the pruning of large colonies is similar to the loss of large colonies due to wave action reported by Birkeland [53] for the sea fan *Gorgonia ventalina*. Populations at many of the sites do not appear to have been resilient to the harvest events, and this correlates with the severity of harvest. Local recruitment as occurs among brooding species can facilitate recovery from disturbance (i.e. Edmunds [54]), but when adult abundance and/or larval survival are too low, effects of the disturbance can generate a negative feedback between abundance and recovery.

## Supporting Information

Table S1 Components of variation in the distribution of *Antillogorgia elisabethae* at 8 sites in The Bahamas in 2004.
(DOCX)

Table S2 Repeated measures ANOVA of ln(1+recruits per quadrat) across site, transects within sites with year as a repeated measure (SPSS, v. 20, Mixed Model Analysis).
(DOCX)

Table S3 Analysis of variance of recruitment of *A. elisabethae* across 8 sites in The Bahamas (SPSS, v.20, Oneway).
(DOCX)

Table S4 Results of Stepwise Linear Regression of ln(1+recruits per quadrat) against Cumulative Adult Area on scales of site, transect, nearest neighbors, and within quadrat and with Regional Density.
(DOCX)

Table S5 Repeated measures analysis of variance of ln(1+ recruits per transect) among sites with before and after harvest as the repeated measure (SPSS v. 20, General Linear Model).
(DOCX)
