# Supplementary material for: Recruitment and Resilience of a Harvested Caribbean Octocoral
Source: PLoS One. 2013 Sep 6;8(9):e74587. doi: 10.1371/journal.pone.0074587 (PMC3765405; doi:10.1371/journal.pone.0074587)
Supplement: Table S1 — Components of variation in the distribution of Antillogorgia elisabethae at 8 sites in The Bahamas in 2004. (DOCX) [file pone.0074587.s001.docx]

Table S1. Components of variation in the distribution of *Antillogorgia elisabethae* at 8 sites in The Bahamas in 2004.

| **Parameter** | **Variance** | **Std. Error** | **% of Total Variance** |
| --- | --- | --- | --- |
| **Recruits (<5 cm tall)** | | | |
| Quadrats | 10.738 | 1.550 | 13.8% |
| Transect (within sites) | 56.831 | 19.662 | 72.8% |
| Site | 10.461 | 16.682 | 13.4% |
| Total | 78.030 |  |  |
|  |  |  |  |
| **Immature (5-20 cm tall)** | | | |
| Quadrats | 4.783 | 0.690 | 55.9% |
| Transect (within sites) | 0.961 | 0.654 | 11.2% |
| Site | 2.814 | 2.089 | 32.9% |
| Total | 8.559 |  |  |
|  |  |  |  |
| **Mature (>20 cm tall)** | | | |
| Quadrats | 3.008 | 0.434 | 75.3% |
| Transect (within sites) | 0.088 | 0.246 | 2.2% |
| Site | 0.897 | 0.679 | 22.5% |
| Total | 3.994 |  |  |
