# Supplementary material for: Recruitment and Resilience of a Harvested Caribbean Octocoral
Source: PLoS One. 2013 Sep 6;8(9):e74587. doi: 10.1371/journal.pone.0074587 (PMC3765405; doi:10.1371/journal.pone.0074587)
Supplement: Table S2 — Repeated measures ANOVA of ln(1+recruits per quadrat) across site, transects within sites with year as a repeated measure (SPSS, v. 20, Mixed Model Analysis). (DOCX) [file pone.0074587.s002.docx]

Table S2 . Repeated measures ANOVA of ln(1+recruits per quadrat) across site, transects within sites with year as a repeated measure (SPSS, v. 20, Mixed Model Analysis).

| **Type III Tests of Fixed Effects** | | | | |
| --- | --- | --- | --- | --- |
| Source | Numerator df | Denominator df | F | Sig. |
| Intercept | 1 | 182.149 | 426.690 | .000 |
| Site | 7 | 125.137 | 120.846 | .000 |
| Year | 3 | 160.682 | 1.655 | .179 |
| Site * Year | 21 | 147.289 | 2.368 | .001 |
| Transect(Site) | 24 | 120.455 | 3.926 | .000 |

Estimated Marginal Means

| Site / Year | | Mean^a^  (ln[1+recruits]) | Std. Error | df | 95% Confidence Interval | |
| --- | --- | --- | --- | --- | --- | --- |
|  |  |  |  |  | Lower Bound | Upper Bound |
| Burrows North | | .304 | .068 | 122.930 | .169 | .438 |
| Burrows South | | .358 | .068 | 122.930 | .224 | .493 |
| Cross Harbour Slope | | 1.611 | .068 | 122.930 | 1.476 | 1.745 |
| Cross Harbour Ridge | | 2.137 | .068 | 122.930 | 2.002 | 2.271 |
| Gorda Patch Reef | | .644 | .068 | 122.930 | .509 | .778 |
| Long Rock | | .036 | .068 | 122.930 | -.099 | .171 |
| Sandy Point 1 | | .187 | .068 | 122.930 | .052 | .321 |
| Sandy Point 2 | | .114 | .189 | 137.560 | -.260 | .488 |
|  | | | | | | |
| 2004 | .642 | | .105 | 113.175 | .434 | .849 |
| 2005 | .757 | | .042 | 130.104 | .674 | .841 |
| 2006 | .666 | | .049 | 133.871 | .569 | .763 |
| 2007 | .630 | | .042 | 128.083 | .548 | .712 |
| ^a^ Based on modified population marginal mean. | | | | | | |
