# Supplementary material for: Recruitment and Resilience of a Harvested Caribbean Octocoral
Source: PLoS One. 2013 Sep 6;8(9):e74587. doi: 10.1371/journal.pone.0074587 (PMC3765405; doi:10.1371/journal.pone.0074587)
Supplement: Table S3 — Analysis of variance of recruitment of A. elisabethae across 8 sites in The Bahamas (SPSS, v.20, Oneway). (DOCX) [file pone.0074587.s003.docx]

Table S3. Analysis of variance of recruitment of *A. elisabethae* across 8 sites in The Bahamas (SPSS, v.20, Oneway).

| **Tests of Between-Subjects Effects** | | | | | |
| --- | --- | --- | --- | --- | --- |
| Dependent Variable: ln(1+recruits per quadrat) | | | | | |
| Source | Type III Sum of Squares | df | Mean Square | F | Sig. |
| Corrected Model | 342.159 | 30 | 11.405 | 28.397 | .000 |
| Intercept | 290.329 | 1 | 290.329 | 722.873 | .000 |
| Site | 325.277 | 7 | 46.468 | 115.699 | .000 |
| Year | 1.387 | 3 | .462 | 1.152 | .328 |
| Site * Year | 15.051 | 20 | .753 | 1.874 | .012 |
| Error | 236.561 | 589 | .402 |  |  |
| Total | 881.699 | 620 |  |  |  |
| Corrected Total | 578.720 | 619 |  |  |  |
| a. R Squared = .591 (Adjusted R Squared = .570) | | | | | |

Scheffe Test for homogenous groups

| Site | N | Means within each Subset | | | |  |
| --- | --- | --- | --- | --- | --- | --- |
|  |  | 1 | 2 | 3 | 4 |  |
| Long Rock | 80 | .0360 |  |  |  |  |
| Sandy Point 1 | 80 | .1869 |  |  |  |  |
| Sandy Point 2 | 60 | .1884 |  |  |  |  |
| Burrows North | 80 | .3037 | .3037 |  |  |  |
| Burrows South | 80 | .3583 | .3583 |  |  |  |
| Gorda Patch Reef | 80 |  | .6438 |  |  |  |
| Cross Harbour Slope | 80 |  |  | 1.6110 |  |  |
| Cross Harbour Ridge | 80 |  |  |  | 2.1367 |  |
| Sig. |  | .195 | .139 | 1.000 | 1.000 |  |
| The error term is Mean Square(Error) =0 .402. | | | | | | |
| Uses Harmonic Mean Sample Size = 76.800. | | | | | | |
| Alpha = 0.05. | | | | | | |
