# Supplementary material for: Recruitment and Resilience of a Harvested Caribbean Octocoral
Source: PLoS One. 2013 Sep 6;8(9):e74587. doi: 10.1371/journal.pone.0074587 (PMC3765405; doi:10.1371/journal.pone.0074587)
Supplement: Table S4 — Results of Stepwise Linear Regression of ln(1+recruits per quadrat) against Cumulative Adult Area on scales of site, transect, nearest neighbors, and within quadrat and with Regional Density. (DOCX) [file pone.0074587.s004.docx]

Table S4. Results of Stepwise Linear Regression of ln(1+recruits per quadrat) against Cumulative Adult Area on scales of site, transect, nearest neighbors, and within quadrat and with Regional Density

| Model | r | r^2^ | Std. Error of the Estimate | Change Statistics | | | | |
| --- | --- | --- | --- | --- | --- | --- | --- | --- |
|  |  |  |  | R Square Change | F Change | df1 | df2 | Sig. F Change |
| 1. Sites* | 0.681 | 0.464 | 0.7254 | 0.464 | 483.740 | 1 | 558 | 0.000 |
| 2. Sites*, Quadrats | 0.696 | 0.484 | 0.7126 | 0.020 | 21.193 | 1 | 557 | 0.000 |
| 3. Sites*, Quadrats, Near-neighbors | 0.701 | 0.492 | 0.7078 | 0.008 | 8.676 |  | 556 | 0.003 |
| 4. Sites*, Quadrats, Near-neighbors, Regional density | .706 | .498 | 0.7046 | 0.006 | 6.833 | 1 | 555 | 0.009 |
| 5. Sites*, Quadrats, Near-neighbors ,Regional density, Transects* | .709 | 0.502 | 0.7019 | 0.004 | 4.407 | 1 | 554 | 0.036 |

| Model 5 Coefficients | Unstandardized Coefficients | | Standardized Coefficients | t | Sig. | 95% Confidence Interval for B | | Colinearity Statistics | |
| --- | --- | --- | --- | --- | --- | --- | --- | --- | --- |
|  | B | Std. Error | β |  |  | Lower Bound | Upper Bound | Tolerance | VIF |
| Constant | .077 | .059 |  | 1.308 | .191 | -.039 | .193 |  |  |
| Sites* | .001 | .000 | .473 | 9.313 | .000 | .001 | .001 | .349 | 2.869 |
| Quadrats | 7.533E-05 | .000 | .137 | 3.852 | .000 | .000 | .000 | .712 | 1.404 |
| Near Neighbors | .000 | .000 | .091 | 2.231 | .026 | .000 | .000 | .535 | 1.870 |
| Regional density | -.001 | .000 | -.079 | -2.618 | .009 | -.002 | .000 | .986 | 1.014 |
| Transect* | .000 | .000 | .102 | 2.099 | .036 | .000 | .000 | .377 | 2.652 |
