# Supplementary material for: Recruitment and Resilience of a Harvested Caribbean Octocoral
Source: PLoS One. 2013 Sep 6;8(9):e74587. doi: 10.1371/journal.pone.0074587 (PMC3765405; doi:10.1371/journal.pone.0074587)
Supplement: Table S5 — Repeated measures analysis of variance of ln(1+ recruits per transect) among sites with before and after harvest as the repeated measure (SPSS v. 20, General Linear Model). (DOCX) [file pone.0074587.s005.docx]

Table S5 - Repeated measures analysis of variance of ln(1+ recruits per transect) among sites with before and after harvest as the repeated measure (SPSS v. 20, General Linear Model)

| **Tests of Within-Subjects Contrasts** | | | | | | |
| --- | --- | --- | --- | --- | --- | --- |
| Source | Type III Sum of Squares | df | Mean Square | F | Sig. | Power^a^ |
| Harvest_Effect | .080 | 1 | .080 | .662 | .424 | .122 |
| Harvest_Effect *Site | 1.496 | 7 | .214 | 1.774 | .139 | .585 |
| Error(Harvest_Effect) | 2.892 | 24 | .121 |  |  |  |

| **Tests of Between-Subjects Effects** | | | | | | |  |
| --- | --- | --- | --- | --- | --- | --- | --- |
| Source | Type III Sum of Squares | df | Mean Square | F | Sig. | Power^a^ | |
| Intercept | 45.360 | 1 | 45.360 | 277.678 | .000 | 1.000 | |
| Site | 52.188 | 7 | 7.455 | 45.640 | .000 | 1.000 | |
| Error | 3.921 | 24 | .163 |  |  |  | |
| a. Computed using alpha = .05 | | | | | | |  |
